# The genetic and clinico‐pathological profile of early‐onset progressive supranuclear palsy

**DOI:** 10.1002/mds.27786

**Published:** 2019-07-12

**Authors:** Edwin Jabbari, John Woodside, Manuela M.X. Tan, Nicola Pavese, Oliver Bandmann, Boyd C.P. Ghosh, Luke A. Massey, Erica Capps, Tom T. Warner, Andrew J. Lees, Tamas Revesz, Janice L. Holton, Nigel M. Williams, Donald G. Grosset, Huw R. Morris

**Affiliations:** ^1^ Department of Clinical and Movement Neurosciences UCL Queen Square Institute of Neurology London United Kingdom; ^2^ Movement Disorders Centre UCL Queen Square Institute of Neurology London United Kingdom; ^3^ Division of Neuroscience Newcastle University Newcastle United Kingdom; ^4^ Sheffield Institute for Translational Neuroscience University of Sheffield Sheffield United Kingdom; ^5^ Wessex Neurological Centre University Hospitals Southampton NHS Foundation Trust Southampton United Kingdom; ^6^ Department of Neurology Poole Hospital NHS Foundation Trust Poole United Kingdom; ^7^ Care of the Elderly Department Shrewsbury and Telford Hospital NHS Trust Shrewsbury United Kingdom; ^8^ Reta Lila Weston Institute UCL Queen Square Institute of Neurology London United Kingdom; ^9^ Queen Square Brain Bank for Neurological Disorders UCL Queen Square Institute of Neurology London United Kingdom; ^10^ Institute of Psychological Medicine and Clinical Neurosciences, Medical Research Council Centre for Neuropsychiatric Genetics and Genomics Cardiff University Cardiff United Kingdom; ^11^ Department of Neurology, Institute of Neurological Sciences Queen Elizabeth University Hospital Glasgow United Kingdom

**Keywords:** clinical neurology, genetics, Parkinson's disease/parkinsonism, progressive supranuclear palsy

## Abstract

**Background:**

Studies on early‐onset presentations of progressive supranuclear palsy (PSP) have been limited to those where a rare monogenic cause has been identified. Here, we have defined early‐onset PSP (EOPSP) and investigated its genetic and clinico‐pathological profile in comparison with late‐onset PSP (LOPSP) and Parkinson's disease (PD).

**Methods:**

We included subjects from the Queen Square Brain Bank, PROSPECT‐UK study, and Tracking Parkinson's study. Group comparisons of data were made using Welch's t‐test and Kruskal‐Wallis analysis of variance. EOPSP was defined as the youngest decile of motor age at onset (≤55 years) in the Queen Square Brain Bank PSP case series.

**Results:**

We identified 33 EOPSP, 328 LOPSP, and 2000 PD subjects. The early clinical features of EOPSP usually involve limb parkinsonism and gait freezing, with 50% of cases initially misdiagnosed as having PD. We found that an initial clinical diagnosis of EOPSP had lower diagnostic sensitivity (33%) and positive predictive value (38%) in comparison with LOPSP (80% and 76%) using a postmortem diagnosis of PSP as the gold standard. 3/33 (9%) of the EOPSP group had an underlying monogenic cause. Using a PSP genetic risk score (GRS), we showed that the genetic risk burden in the EOPSP (mean *z‐*score, 0.59) and LOPSP (mean *z‐*score, 0.48) groups was significantly higher (*P* < 0.05) when compared with the PD group (mean *z‐*score, −0.08).

**Conclusions:**

The initial clinical profile of EOPSP is often PD‐like. At the group level, a PSP GRS was able to differentiate EOPSP from PD, and this may be helpful in future diagnostic algorithms. © 2019 The Authors. *Movement Disorders* published by Wiley Periodicals, Inc. on behalf of International Parkinson and Movement Disorder Society.

Progressive supranuclear palsy (PSP) is a neurodegenerative disease and common cause of atypical parkinsonism, with an estimated prevalence of 5 to 7 per 100,000.^1^ The neuropathology of PSP, the gold standard for diagnosis, is centred on the structural microtubule‐associated protein tau, encoded by the *MAPT* gene.^2^

A comprehensive, case‐control genome‐wide association study has shown that loci at *MAPT* (H1 haplotype and H1c subhaplotype), *MOBP*, *STX6,* and *EIF2AK3* are associated with PSP.^3^

The clinical heterogeneity of PSP has been highlighted by the description of Richardson's syndrome (RS)^4^, ^5^ and other syndromes with identical pathology including PSP‐parkinsonism (PSP‐P) and PSP‐pure akinesia with gait freezing (PSP‐PGF),^6^, ^7^ which are included in recent Movement Disorder Society (MDS) diagnostic criteria.^8^ This heterogeneity is associated with common variation at the *TRIM11* locus^9^ and variability in the regional distribution and density of pathogenic tau accumulation.^10^

Studies involving younger patients with PSP have focused on familial cases with an identified single gene mutation despite the fact that PSP is considered to be a sporadic disease.^11^ Studies of autosomal‐dominant familial PSP phenotypes with variable disease durations and PSP‐type tau pathology at postmortem examination have identified *MAPT* and *LRRK2* mutations.^11^ Of note, monogenic PSP mimics in younger patients include Perry syndrome (*DCTN1* mutations) and Niemann‐Pick type C (*NPC1/NPC2)* mutations.^11^, ^12^

In this study, we define early‐onset PSP (EOPSP), clarify its differential diagnosis, describe its clinico‐pathological profile, and identify potential determinants of early‐onset disease.

## Methods

### Patient Consent

All patients gave written informed consent for the use of their medical records and brain tissue/blood samples for research purposes, including the analysis of DNA.

All subjects, regardless of their pathological diagnosis, who had an antemortem clinical diagnosis of PSP at any point in their disease course were identified from the Queen Square Brain Bank (QSBB), with the year of death ranging from 2000 to 2018. The brain donor program was approved by a London multicenter research ethics committee, and tissue is stored for research under a license from the Human Tissue Authority, No. 12198. To enable the calculation of diagnostic sensitivity and positive predictive value (PPV), we later included patients with a postmortem diagnosis of PSP where PSP was not the initial or final clinical diagnosis.

Progressive Supranuclear Palsy‐Corticobasal Syndrome‐Multiple System Atrophy‐UK (PROSPECT‐UK) is a U.K.‐wide longitudinal study of patients with atypical parkinsonian syndromes, including PSP (Queen Square Research Ethics Committee 14/LO/1575). Subjects with a baseline clinical diagnosis of PSP from the PROSPECT‐UK study were identified, with the year of recruitment ranging from 2015 to 2018.^13^

Tracking Parkinson's is a U.K.‐wide longitudinal study of Parkinson's disease (PD) across 72 sites, with multicenter ethics committee and local research and development department approvals. Subjects with a baseline clinical diagnosis of PD were identified, with the year of recruitment ranging from 2012 to 2014.^14^ PD cases were diagnosed consistent with QSBB clinical diagnostic criteria.^15^

### Defining EOPSP

EOPSP was defined in patients with a clinical diagnosis of PSP, consistent with MDS clinical diagnostic criteria,^8^ and a motor symptom onset ≤55 years of age. This threshold was used as it represented the youngest decile of age at motor symptom onset in the QSBB series of pathologically diagnosed PSP cases. Late‐onset PSP (LOPSP) was defined as cases with a clinical diagnosis of PSP and a motor symptom onset >55 years of age.

### Clinical Data Collection and Phenotyping

The following clinical features were recorded for each case: sex, ethnicity, family history of dementia and/or parkinsonism in first‐degree relatives, age at motor symptom onset, initial clinical diagnosis and PSP phenotype, final/current clinical diagnosis and PSP phenotype, diagnostic latency (from motor symptom onset to correct diagnosis), and disease duration (from motor symptom onset to death) in deceased cases. Of note, the initial clinical diagnosis/phenotype was defined as the clinical diagnosis/MDS criteria PSP phenotype given to patients in the first 3 years after their motor symptom onset. Final/current clinical diagnosis/phenotype was defined as the clinical diagnosis/MDS criteria PSP phenotype given to patients at least 2 years after the date of their initial clinical diagnosis/phenotype. Although we emphasize the age at motor symptom onset to define EOPSP/LOPSP, we screened each case for the onset and burden of cognitive symptoms relative to motor symptom onset to identify patients with frontal presentations of PSP.

In conjunction with the phenotyping methods described above, the presence or absence of MDS PSP criteria clinical features^8^ in the initial and final/current disease stages were used to produce radar charts to further highlight the phenotypic differences between EOPSP, LOPSP, and PD.

PROSPECT‐UK subjects had serial PSP rating scale (PSPRS) scores recorded, and both PROSPECT‐UK and Tracking Parkinson's subjects had serial MDS‐UPDRS part III scores recorded to assess the rates of clinical disease progression.

To compare the rates of encephalitis and head injury between the EOPSP and LOPSP cases, we collected data on the presence or absence of a documented past medical history of encephalitis in case notes (QSBB cases only) and the presence or absence of any mode of head injury resulting in loss of consciousness prior to the onset of PSP motor symptoms, identified from the Retrospective Screening of Traumatic Brain Injury (RESTBI) questionnaire (PROSPECT‐UK cases only).

### Neuropathological Diagnosis

The neuropathological examinations of EOPSP and LOPSP cases in this study were carried out at QSBB by J.L.H. and T.R. The pathological diagnoses of these cases were used to calculate the diagnostic sensitivity and PPV of EOPSP and LOPSP using a pathological diagnosis of PSP as the gold standard. Neuropathological data from Tracking Parkinson's PD cases was not available for analysis. 

### Genotyping

PSP and PD cases had DNA extracted from either brain tissue or blood. We then genotyped DNA samples using the Illumina (San Diego, CA) NeuroChip^16^ for all QSBB and PROSPECT‐UK PSP cases, and the Illumina Human Core Exome array^14^ for all Tracking Parkinson's PD cases. Importantly, none of the PSP cases in this study had been included in the original PSP case‐control genome‐wide association study.^3^ Standard genotype data quality control steps were carried out.^17^ All cases were screened for the known pathogenic *MAPT, LRRK2, PRKN, PINK1, SNCA, GRN,* and *DCTN1* mutations, which are directly genotyped on both the Illumina NeuroChip and Illumina Human Core Exome Array (detailed list of pathogenic variants can be found in the supplementary data of Blauwendraat et al.^16^). Standard quality control steps for single nucleotide polymorphism imputation were carried out.^17^ *MAPT* H1/H1 frequency (determined by rs1800547 genotype), *TRIM11* minor allele frequency (determined by rs564309 genotype), and *APOE* ε4 allele frequency (determined by rs429358 and rs7412 genotypes) were extracted for all cases. In addition, one biochemically proven case of Niemann‐Pick type C disease had targeted sequencing to identify pathogenic mutations in the *NPC1* and *NPC2* genes.

### Statistical Analyses

All statistical analyses were carried out using Stata version 15 (StataCorp. 2017. Stata Statistical Software: Release 15. College Station, TX: StataCorp LLC) and Plink version 1.9 (Harvard University, Cambridge, MA). Figures were generated using R version 3.3.2 (R Foundation for Statistical Computing, Vienna, Austria). Statistical significance was defined as *P* < 0.05.

We studied the pathological diagnoses and/or genetic mutation status of the EOPSP, LOPSP, and PD cases. Cases with alternative pathological and/or genetic diagnoses were excluded from subsequent analyses of clinical and genetic data. Group comparisons of clinical features were made using Welch's t‐test.

Clinically diagnosed PSP and PD cases with an alternative current clinical diagnosis were excluded from our genetic analyses. A PSP genetic risk score (GRS), based on weighted odds ratios for all risk loci (*MAPT* H1 haplotype and H1c subhaplotype, *MOBP*, *STX6,* and *EIF2AK3*) identified in the original PSP case‐control genome‐wide association study,^3^ was calculated for all white PSP and PD cases (confirmed by principal component analysis). Group comparisons of PSP GRS *z‐*scores were made using Kruskal‐Wallis analysis of variance.

We used t‐testing to look for clinical and genetic differences between QSBB and PROSPECT‐UK cases within our EOPSP and LOPSP groups.

We collected the following data from QSBB cases to compare the diagnostic sensitivity and PPV of initial and final clinical diagnoses of EOPSP and LOPSP in our cohort: (1) the primary pathological diagnosis of all cases with initial and/or final clinical diagnoses of EOPSP/LOPSP; (2) the initial and final clinical diagnoses of all cases with a primary pathological diagnosis of PSP, which includes cases that never had an antemortem diagnosis of PSP.

### Variable Age at Onset to Define EOPSP

Alongside our arbitrary age at onset cut‐off point (youngest 10% of QSBB PSP series = ≤55 years) to define EOPSP, we assessed the impact of changing the age at onset cut‐off point on the clinical profile, PSP GRS z‐score, and diagnostic sensitivity/PPV of EOPSP in comparison with LOPSP. Specifically, the alternative age at onset cut‐off points studied were as follows: (1) youngest 5% of QSBB PSP series = ≤52 years; (2) youngest 15% of QSBB PSP series = ≤59 years; (3) youngest 20% of QSBB PSP series = ≤62 years; (4) youngest 25% of QSBB PSP series = ≤64 years. The definition of LOPSP varied with each alternative age at onset cut‐off point accordingly.

### Data Availability

The raw data used for analyses in this study will be considered for sharing in anonymized format by request of a qualified investigator to the corresponding authors for purposes of replicating the procedures and results.

## Results

We identified 361 subjects with a clinical diagnosis of PSP at any point in their disease course from the QSBB and PROSPECT‐UK study with detailed clinical data available throughout the entire disease course. Of the PSP cases, 33/361 (9%) fulfilled criteria for EOPSP. In addition, 2000 PD cases from the Tracking Parkinson's study were included. The neuropathological and genetic mutation status of our groups are summarized in Table 1. All PSP subjects with alternative pathological diagnoses were excluded from subsequent analyses. In our EOPSP group, two cases had *MAPT* mutations that have previously been reported in subjects with PSP pathology.^18^, ^19^ One EOPSP case had previously described pathogenic *NPC1* mutations^20^ and was therefore excluded from subsequent analyses. A total of 21 PD cases had *LRRK2*, *PRKN*, and *SNCA* mutations previously reported in subjects with PD pathology.^21^

**Table 1:** Neuropathological and genetic mutation status of study cohort

Feature	EOPSP	LOPSP	PD
No. of subjects	33	328	2000
Postmortem cohort with a pathological diagnosis of PSP, n/N (% of postmortem cohort)	14/20 (70)	129/158 (82)	
Postmortem cohort with alternative pathological diagnoses, n/N (% of postmortem cohort)	AD: 2/20 (10) PD: 2/20 (10) CBD: 1/20 (5) MSA: 1/20 (5)	CBD: 10/158 (6) PD: 5/158 (3) AD: 4/158 (3) MSA: 4/158 (3) ALS: 2/158 (1) PiD: 2/158 (1) AGD: 2/158 (1)	
Cases with a pathogenic genetic mutation, n/N (% of whole group)	3/33 (9)	0/328 (0)	21/1566 (1)
Pathogenic genetic mutations (number of cases)	*MAPT* IVS10 + 16 (1) *MAPT* L284R (1) *NPC1* heterozygous c.1844G > T p.(Arg615Leu) and *NPC1* heterozygous c.3182T > C p.(IIe1061Thr) (1)	None	*LRRK2* G2019S (17) *LRRK2* R1441C heterozygous (2) *PRKN* p.P113Xfs/*PRKN *exon 5 hemizygous deletion (compound heterozygous) (1) *SNCA* heterozygous duplication of exons 1‐6 (1)

EOPSP, early‐onset PSP; LOPSP, late‐onset PSP; PD, Parkinson's disease; PSP, progressive supranuclear palsy; AD, Alzheimer's disease; CBD, corticobasal degeneration; MSA, multiple system atrophy; ALS, amyotrophic lateral sclerosis; PiD, Pick's disease; AGD, argyrophilic grain disease; *MAPT*, microtubule‐associated protein tau; *NPC1*, Niemann‐Pick type C1; *LRRK2*, leucine‐rich repeat kinase 2; *PRKN*, Parkin; *SNCA*, alpha synuclein.

**Figure 1:**
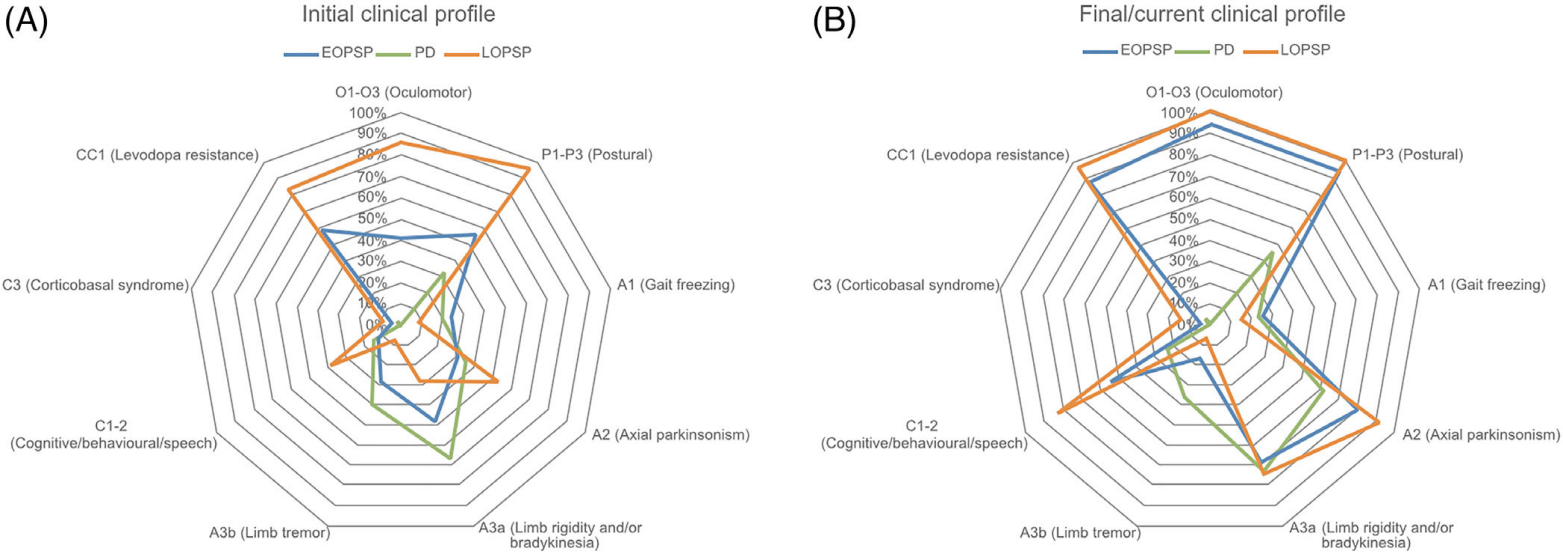
Initial (**A**) and final/current (**B**) clinical profiles of EOPSP, LOPSP, and PD. Radar charts comparing the percentage (%) of EOPSP, LOPSP, and PD cases with Movement Disorder Society PSP diagnostic criteria clinical features in early (A) and late (B) stages of disease. EOPSP, early‐onset PSP; LOPSP, late‐onset PSP; PD, Parkinson's disease; PSP, progressive supranuclear palsy.

The clinical profiles of our groups are summarized in Table 2. The initial clinical profile of EOPSP was more PD‐like when compared with LOPSP (Fig. 1). The final/current clinical profiles of EOPSP and LOPSP both resembled RS (Fig. 1). Deceased EOPSP cases had a longer disease duration than deceased LOPSP and PD cases (Table 2). However, only 5% of the Tracking Parkinson's study cohort were deceased at the point of censoring, so a majority of these deceased PD cases are likely to be atypical fast progressing cases, with no neuropathological data available. In comparison with an age‐matched (motor symptom onset ≤55 years) cohort of PD cases from the Tracking Parkinson's study (n = 328, mean age at onset = 48.5 years), our EOPSP group had a significantly longer mean diagnostic latency (3.2 vs. 2.5 years; *P* < 0.05).

**Table 2:** Clinical profile of study cohort

Feature	EOPSP	LOPSP	PD
No. of subjects	26	299	2000
% male	69	62	65
Ethnicity (% of cases)	CEU (92) Non‐CEU (8)	CEU (92) Non‐CEU (8)	CEU (97) Non‐CEU (3)
Family history of dementia and/or parkinsonism, % of cases	27	15	22
Age at motor symptom onset, yr–mean (SD), range	51.0^a^, ^b^, ^c^ (4.8) 40–55	68.1^c^ (6.3) 56–88	64.4 (9.7) 23–90
Initial clinical diagnosis (% of cases)	PD (50) PSP (31) VascP (7) Dementia (4) CBS (4) ET (4)	PSP (80) PD (10) CBS (7) FTD (2) NPH (1)	PD (100)
Initial PSP phenotype (% of cases)	s.o. PSP‐P (31) prob. PSP‐RS (27) poss. PSP‐PGF (19) prob. PSP‐P (15) s.o. PSP‐F (4) s.o. PSP‐CBS (4)	prob. PSP‐RS (64) prob. PSP‐P (12) poss. PSP‐PGF (8) s.o. PSP‐CBS (7) s.o. PSP‐P (6) s.o. PSP‐F (3)	
Final/current clinical diagnosis (% of cases)	PSP (96) CBS (4)	PSP (91) CBS (5) MSA (1) FTD (1) LBD (1) APS (1)	PD (98) SWEDD (0.4) MSA (0.3) PSP (0.2) Other^d^ (1.1)
Final/current PSP phenotype (% of cases)	prob. PSP‐RS (84) prob. PSP‐P (8) prob. PSP‐PGF (4) s.o. PSP‐CBS (4)	prob. PSP‐RS (84) s.o. PSP‐CBS (5) prob. PSP‐P (4) poss. PSP‐CBS (3) prob. PSP‐PGF (2) prob. PSP‐F (2)	
Diagnostic latency, yr–mean (SD)	3.2^a^, ^b^, ^c^ (1.5)	2.2^c^ (1.3)	1.8 (2.8)
% of cases deceased	69	56	5
Disease duration in deceased cases, yr–mean (SD), range	10.5^a^, ^b^, ^c^ (3.9) 4.4–15.2	6.2^c^ (2.6) 2.4–15.9	6.0 (5.0) 2.3–45.2

Group comparisons made using Welch's t‐test.

a *P* < 0.05 vs. PD.

b *P* < 0.05 vs. LOPSP.

c No significant intra‐group difference between Queen Square Brain Bank and PROSPECT‐UK cases.

d Other diagnoses consist of essential tremor, corticobasal syndrome, dystonic tremor, functional neurological disorder, multiple sclerosis, and vascular parkinsonism.

EOPSP, early‐onset PSP; LOPSP, late‐onset PSP; PD, Parkinson's disease; CEU, Caucasian residents of European ancestry from Utah; SD, standard deviation; PSP, progressive supranuclear palsy; VascP, vascular parkinsonism; CBS, corticobasal syndrome; ET, essential tremor; FTD, frontotemporal dementia; NPH, normal pressure hydrocephalus; s.o., suggestive of; prob., probable; poss., possible; PSP‐P, PSP‐parkinsonism; PSP‐RS, PSP‐Richardson's syndrome; PSP‐PGF, PSP‐pure akinesia with gait freezing; PSP‐F, PSP‐frontal; PSP‐CBS, PSP‐corticobasal syndrome overlap; LBD, Lewy body dementia; APS, atypical parkinsonian syndrome; SWEDD, scans without evidence of dopaminergic deficit; MSA, multiple system atrophy.

None of the QSBB EOPSP or LOPSP cases had a past medical history of encephalitis. Of the PROSPECT‐UK EOPSP cases, 1/12 (8%) reported experiencing a head injury that resulted in a loss of consciousness prior to the onset of PSP motor symptoms compared with 7/183 (4%) of the PROSPECT‐UK LOPSP cases. The mean latency of time from concussive head injury to onset of PSP motor symptoms was 44.5 years in the EOPSP group and 38.9 years in the LOPSP group.

A total of 1878 cases had genotyping data available that passed quality control thresholds to be included in genetic analyses. Of note, the two EOPSP *MAPT* mutation cases were excluded from these analyses. The minor allele frequency of the *TRIM11* rs564309 single nucleotide polymorphism was higher in the EOPSP group in comparison with the LOPSP group, coinciding with a higher rate of non‐RS phenotypes in EOPSP. We found significantly higher PSP GRS *z‐*scores in the EOPSP and LOPSP groups when compared with the PD group, with no significant differences between the EOPSP and LOPSP groups (Table 3). Summary statistics for individual PSP risk loci that were included in the PSP GRS are available online (Supporting Information Table 1).

**Table 3:** Genetic profile of study cohort

Feature	EOPSP	LOPSP	PD
No. of subjects	24	288	1566
*MAPT* H1/H1 frequency, %	92	90	67
*APOE* ε4 allele frequency, %	18	10	13
*TRIM11* rs564309 MAF, %	21	11	15
PSP GRS *z‐*score, mean (SE)	0.59^a^, ^b^ (0.13)	0.48^a^, ^b^ (0.05)	−0.08 (0.03)

Group comparisons of PSP GRS z‐scores made using Kruskal‐Wallis analysis of variance.

a *P* < 0.05 vs. PD.

b No significant intra‐group difference between Queen Square Brain Bank and PROSPECT‐UK cases.

EOPSP, early‐onset PSP; LOPSP, late‐onset PSP; PD, Parkinson's disease; *MAPT*, microtubule‐associated protein tau; *APOE*, apolipoprotein E; *TRIM11*, tripartite motif‐containing protein 11; MAF, minor allele frequency; PSP, progressive supranuclear palsy; GRS, genetic risk score; SE, standard error.

A subset of PROSPECT‐UK EOPSP (n = 5) and LOPSP (n = 42) cases, and Tracking Parkinson's PD (n = 570) cases, had baseline and 2‐year or 3‐year PSPRS and/or MDS‐UPDRS part III scores recorded. There were similar mean baseline clinical rating scale scores between EOPSP (PSPRS = 34.2, MDS‐UPDRS part III = 33.2) and LOPSP (PSPRS = 33.4, MDS‐UPDRS part III = 37.5) groups, although the mean disease duration at baseline testing in the EOPSP group was higher in comparison with the LOPSP group (4.1 years vs. 2.6 years). We found no significant differences in the subscale (history, mentation, bulbar, ocular, limb, and gait) scores of the baseline PSPRS between the EOPSP and LOPSP groups. The mean baseline MDS‐UPDRS part III score was significantly higher in the LOPSP group when compared with the PD group (37.5 vs. 20.9; *P* < 0.05). We found that there was a non‐significant trend toward the mean annualized change in PSPRS being lower in the EOPSP group when compared with the LOPSP group (+9.9 vs. +12.8). In contrast, the mean annualized change in MDS‐UPDRS part III scores were significantly lower in the PD group when compared with both EOPSP and LOPSP groups (+3.3 vs. +19.6 and +14.9; *P* < 0.05), with no significant difference between the EOPSP and LOPSP groups.

Using a pathological diagnosis of PSP as the gold standard, we reviewed 160 QSBB cases and found that an initial clinical diagnosis of EOPSP had a lower diagnostic sensitivity (33%) and PPV (38%) in comparison with an initial clinical diagnosis of LOPSP (80% and 76%). In contrast, the diagnostic sensitivity and PPV of a final clinical diagnosis of EOPSP (93% and 76%) were higher and similar to that of LOPSP (89% and 84%).

We carried out analyses to investigate the impact of using alternative age at onset cut‐off points to define EOPSP (Table 4). This revealed that as the age at onset cut‐off point was increased, the EOPSP and LOPSP groups became more homogeneous in their clinical phenotype and PSP GRS *z‐*scores.

**Table 4:** Impact of alternative age at onset cut‐off points to define EOPSP

	EOPSP					LOPSP				
Age‐at‐onset cut‐off, yr^a^	≤52	≤55	≤59	≤62	≤64	>52	>55	>59	>62	>64
No. of subjects	12	26	49	94	117	313	299	276	231	208
Cases with PSP‐P/PSP‐PGF initial clinical phenotype, %	67	65	39	29	26	22	26	21	21	22
Mean PSP GRS *z‐*score	0.57	0.59	0.37	0.40	0.44	0.50	0.48	0.52	0.53	0.52
Sensitivity, %^b^	20	33	50	63	71	81	80	83	84	82
PPV, %^b^	33	38	53	56	61	75	76	77	81	82

Analysis on the impact of alternative age at onset cut‐off points to define EOPSP.

a Age at onset cut‐off points represent the 5%, 10%, 15%, 20%, and 25% age at onset percentile cut‐off points from the Queen Square Brain Bank PSP case series.

b Analyses restricted to only cases with a pathological diagnosis from the Queen Square Brain Bank.

EOPSP, early‐onset PSP; LOPSP, late‐onset PSP; PSP‐P, PSP‐parkinsonism; PSP‐PGF, PSP‐pure akinesia with gait freezing; PSP, progressive supranuclear palsy; GRS, genetic risk score; PPV, positive predictive value.

## Discussion

This is the first study to define EOPSP and describe its genetic and clinico‐pathological profile. Using our definition of an age at motor symptom onset ≤55 years of age, we found that up to 10% of PSP cases were early‐onset in nature. A similar frequency of cases has been observed in young‐onset PD (defined as age at onset <50 years of age^22^) and young‐onset multiple system atrophy (defined as age at onset <40 years of age^23^).

Our study highlights a number of important points that are relevant to both clinical and research settings. First, we show the value of screening patients presenting with an EOPSP syndrome for PSP mimics and rare genetic mutations known to cause familial PSP pathology. Of note, higher rates of a family history of dementia and/or parkinsonism were noted in the EOPSP group when compared with the LOPSP group, even when we discount the two identified *MAPT* mutation EOPSP cases. This observation has been noted in PSP previously^24^ and suggests that there may be novel genetic causes of familial PSP that have yet to be identified.

The overall diagnostic sensitivity and PPV of an initial clinical diagnosis of EOPSP was considerably lower than that of LOPSP. The diagnostic sensitivity and PPV of a final clinical diagnosis of EOPSP and LOPSP were predictably higher and similar to values obtained by Hughes and colleagues.^25^

In comparison with LOPSP and PD, a clinical diagnosis of EOPSP had a longer diagnostic latency; 50% of our EOPSP cases were initially misdiagnosed as PD, and this coincided with the initial clinical profile of EOPSP being dominated by limb parkinsonism and gait freezing. The most common initial MDS PSP criteria phenotypes in association with these presentations were “suggestive of” PSP‐P and “possible” PSP‐PGF. In these cases, although abnormal eye movements had yet to occur to permit “probable” PSP‐P/PSP‐PGF diagnoses, the presence of early postural instability and/or progressive gait freezing (in the context of parkinsonism) were key clinical features that enabled the differentiation of EOPSP from PD.

The final clinical profile of EOPSP closely resembled that of LOPSP and mirrored previous studies which have shown that initial non‐RS phenotypes come to resemble RS in the latter stages of disease.^26^ As we move into a new era of potential anti‐tau therapies,^27^ early and accurate distinction between PSP and PD will become increasingly important. Therefore, we explored the value of a PSP GRS and found that, at the group level, EOPSP and LOPSP *z‐*scores were significantly higher in comparison with PD.

In the absence of an identified genetic mutation, the biological drivers of early‐onset presentations of neurodegenerative diseases such as PSP are likely to be multifactorial. We explored the potential aetiological roles of encephalitis and head injury and found no significant differences in rates between EOPSP and LOPSP.

Our findings of a longer disease duration in deceased EOPSP cases in comparison with LOPSP is consistent with a previous clinico‐pathological study that compared early‐onset and late‐onset PD subjects.^28^ One potential explanation for this is the fact that our EOPSP group mostly consisted of cases with PSP‐P and PSP‐PGF phenotypes, which have been associated with slower rates of disease progression.^6^, ^7^, ^26^ However, another possibility, outside the scope of this study, is the likelihood of lower rates of co‐pathologies in EOPSP cases, with a similar study demonstrating an increased age at death in neurodegenerative diseases with minimal co‐pathology.^29^

One of the major strengths of our study was the in‐depth clinical data that was available to us from both the QSBB case notes and serial clinical assessments of patients in the PROSPECT‐UK and Tracking Parkinson's studies. Within the EOPSP and LOPSP groups, the similarity in clinico‐genetic profiles between the QSBB and PROSPECT‐UK cases suggest that our findings are robust and that the diagnostic accuracy of our PROSPECT‐UK EOPSP and LOPSP cases is high. Similar to previous early‐onset studies of movement disorders,^22^, ^23^ we used an arbitrary age at onset cut‐off point to define EOPSP. However, in our study we have gone further by investigating the impact of changing the EOPSP age at onset cut‐off point. This approach was particularly useful as it further justified our initial approach of using an age at onset cut‐off point of ≤55 years of age to define EOPSP. When compared with the other cut‐off points studied, a cut‐off point of ≤55 years of age highlighted the greatest difference in clinical phenotype and PSP GRS *z*‐scores between the EOPSP and LOPSP groups.

The main limitation of this study was the relatively small number of EOPSP cases that were available for analysis. In addition, our genetic mutation rates in EOPSP are based on pathogenic mutations that are directly genotyped on the Illumina NeuroChip, and targeted *NPC1* and *NPC2* sequencing was limited to cases that had biochemical evidence of Niemann‐Pick type C disease. A more accurate estimation of pathogenic genetic mutation rates will be achieved by carrying out whole‐genome sequencing of our cases. We also acknowledge that the absence of pathological confirmation in our large PD cohort may lead to the inclusion of non‐PD patients. However, this is likely to be applicable to a very small number of patients as previous studies have shown that the PPV of a clinical diagnosis of PD going on to have pathological confirmation of PD is as high as 98.6%.^25^

In conclusion, EOPSP was defined as cases with a clinical diagnosis of PSP and a motor symptom onset ≤55 years of age. Genetic testing for familial *MAPT* mutations and PSP mimics is recommended in this patient group. The diagnostic accuracy of EOPSP is lower than that of LOPSP in the early stages of disease, and this coincides with the initial clinical profile of EOPSP being similar to PD. At the group level, a PSP GRS was able to differentiate EOPSP from PD, and this may be helpful in future diagnostic algorithms.

## Author Roles

1) Research project: A. Conception, B. Organization, C. Execution; 2) Statistical Analysis: A. Design, B. Execution, C. Review and Critique; 3) Manuscript: A. Writing of the first draft, B. Review and Critique.

E.J.: 1A, 1B, 1C, 2A, 2B, 2C, 3A, 3B

J.W.: 1B, 1C, 2A, 2C

M.M.X.T.: 1C, 2A, 2B, 2C

N.P.: 1C

O.B.: 1C

B.C.P.G.: 1C

L.A.M.: 1C

E.C.: 1C

T.T.W.: 1B, 1C, 3B

A.J.L.: 1B, 1C, 3B

T.R.: 1C

J.L.H.: 1C

N.M.W.: 1C, 2B, 2C, 3B

D.G.G.: 1C, 2C, 3B

H.R.M.: 1A, 1B, 1C, 2A, 2C, 3B

## Full financial disclosures for the past 12 months

Nothing to report.

## Supporting information


**Supplementary Table 1** PSP risk loci
