# Supplementary material for: The genetic and clinico‐pathological profile of early‐onset progressive supranuclear palsy
Source: Mov Disord. 2019 Jul 12;34(9):1307–14. doi: 10.1002/mds.27786 (PMC6790973; doi:10.1002/mds.27786)
Supplement: Supplementary file 1 — Supplementary Table 1 PSP risk loci [file MDS-34-1307-s001.docx]

**Supplementary table 1:** PSP risk loci

|  | **EOPSP** | **LOPSP** | **PD** | **Controls*** |
| --- | --- | --- | --- | --- |
| **No. of**  **subjects** | 24 | 288 | 1566 | 3287 |
| ***MAPT* H1 haplotype – rs8070723 MAF** | 0.04 | 0.06 | 0.18 | 0.23 |
| ***MAPT* H1c sub-haplotype – rs242557 MAF** | 0.50 | 0.45 | 0.40 | 0.35 |
| ***MOBP* – rs1768208 MAF** | 0.35 | 0.33 | 0.28 | 0.29 |
| ***STX6* – rs1411478 MAF** | 0.44 | 0.44 | 0.39 | 0.42 |
| ***EIF2AK3* – rs7571971 MAF** | 0.40 | 0.34 | 0.28 | 0.26 |

*EIF2AK3* = eukaryotic translation initiation factor 2 alpha kinase 3, EOPSP = early onset PSP, GRS = genetic risk score, LOPSP = late onset PSP, *MAPT* = microtubule associated protein tau, *MOBP* = myelin associated oligodendrocyte basic protein, PD = Parkinson’s disease, PSP = progressive supranuclear palsy, MAF = minor allele frequency, *STX6* = syntaxin 6.

***** Controls data taken from Hoglinger et al. stage 1 PSP case-control GWAS **(3)**.
